# A CARE-compliant article: a case report of possible association between recurrence of multiple evanescent white dot syndrome and the Herpesviridae family

**DOI:** 10.1097/MD.0000000000019794

**Published:** 2020-04-10

**Authors:** Yu-Lin Haw, Teng-Chieh Yu, Chang-Sue Yang

**Affiliations:** a Department of Medicine, Fu Jen Catholic University; b Department of Ophthalmology, Shin Kong Wu Ho-Su Memorial Hospital; c Department of Ophthalmology, School of Medicine, Taipei Medical University, Taipei, Taiwan.

**Keywords:** Herpesviridae, multiple evanescent white dot syndrome, recurrence, retinitis

## Abstract

**Rationale:**

Multiple evanescent white dot syndrome (MEWDS) is a self-limited multifocal chorioretinopathy that typically affects otherwise healthy young females in the second to fourth decades of life. Current understanding of the pathophysiology of MEWDS is still limited. One of the possible underlying causes is an infectious etiology.

**Patient concerns:**

A 24-year-old female with recurrent episodes of typical MEWDS ocular manifestation was observed over 2 years. Viral-specific antibody serologic tests showed evidence of exposure to the Herpesviridae family during the acute stage of MEWDS in the first and recurrent episodes.

**Diagnoses:**

MEWDS was diagnosed by the clinical findings and ancillary testing results of fundus photography, optical coherence tomography, fluorescein angiography, indocyanine green angiography and electroretinogram. The laboratory serology data was positive for varicella-zoster virus (VZV) immunoglobulin M (IgM) in the first episode and exhibited high Epstein–Barr virus (EBV) elevated immunoglobulin G (IgG) titer in the recurrent episode.

**Interventions:**

Due to the self-limited nature of MEWDS, we observed the clinical course without intervention.

**Outcomes:**

During acute onset of MEWDS, serologic data for VZV IgM antibody was positive in the first episode. Two years later, the patient had recurrent episodes of MEWDS in the contralateral eye. Serologic study showed highly elevated IgG titer (1:160) of Epstein-Barr virus capsid antigen (EB-VCA) in the acute stage. The follow-up paired serum virus serology test showed that the prior EB-VCA IgG titer decreased fourfold to 1:40 in the recovery stage.

**Lessons:**

Recurrence of MEWDS may be associated with acute systemic infection of the Herpesviridae family or virus-induced autoimmune inflammatory reaction.

## 1 Introduction

Multiple evanescent white dot syndrome (MEWDS) was first described in 1984 by Jampol et al as an idiopathic, multifocal, chorioretinopathy. It affects females more than males, with a ratio of 5:1.^[1]^ These patients are typically healthy and in their second to fourth decades of life. Roughly, half of the patients affected by this disease state that they had a prodromal flu-like illness preceding their ocular complaints of blurred vision, shimmering photopsias, dyschromatopsia, and a paracentral and often temporal scotoma.^[2]^ While MEWDS is mostly a unilateral process, bilateral cases of MEWDS have been described.^[3]^ On examination, visual acuity may vary from 20/20 to 20/400, and a relative afferent pupillary defect may be present. The anterior segment is often void of signs of inflammation; however, mild vitreous cells are often present. The optic nerve may be hyperemic. The characteristic, multiple, ill-defined, yellow-white dots are located at the level of the retinal pigment epithelium (RPE) or outer retina and are distributed predominantly in the perimacular area and extend out to the mid-peripheral retina. Classical foveal granularity appearance is observed.^[4]^ Optical coherence tomography (OCT) displays subtle disruptions of the ellipsoid zone (EZ) of the photoreceptor cells in the acute inflammatory stage.^5^,^6^ These ocular signs usually spontaneously resolve within weeks to months, and recurrence is rare.

This paper reports 1 typical case of recurrent MEWDS. Ocular findings, multimodal imaging, and serological data are described. The pathogenesis of recurrent MEWDS is still unclear. This report investigates and proposes the association between recurrent MEWDS and the Herpesviridae family, which may provide clues for predicting whether patients with MEWDS are likely to suffer from recurrent episodes. It also discusses the possible pathogenesis pathway of MEWDS recurrence in the same eye or the contralateral eye.

## 2 Case report

A 24-year-old Chinese female presented with persistent flashes of light and blurred vision in the left eye, which she had experienced for 10 days. She had contracted an upper respiratory tract infection a week before. Otherwise, she had no medical history of systemic illness or recent vaccinations. No systemic varicella or prior vaccinations of varicella-zoster virus (VZV) were noted before this episode. The visual acuity of both eyes was 6/5. The intraocular pressure (IOP) was 22 mmHg in the right eye and 18 mmHg in the left eye. Examinations of the anterior chamber and vitreous revealed no cells or flares.

**Figure 1:**
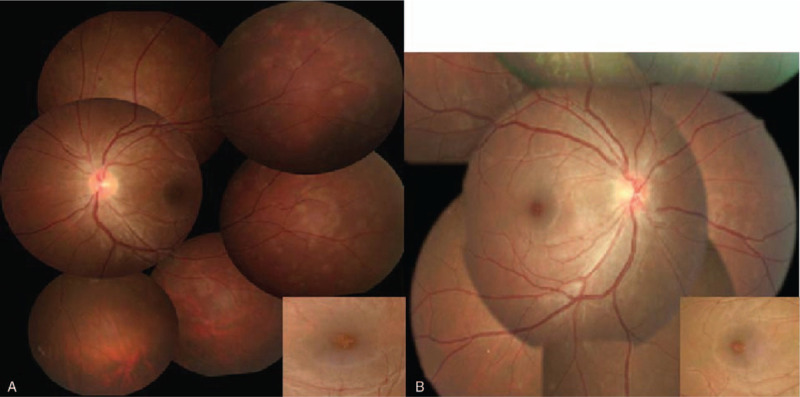
Fundus photography showed numerous white dots over the macula to the mid-peripheral fundus, with characteristic yellowish foveal granularity (inset). (A) The first episode occurring in the left eye. (B) The recurrent episode occurring in the right eye.

**Figure 2:**
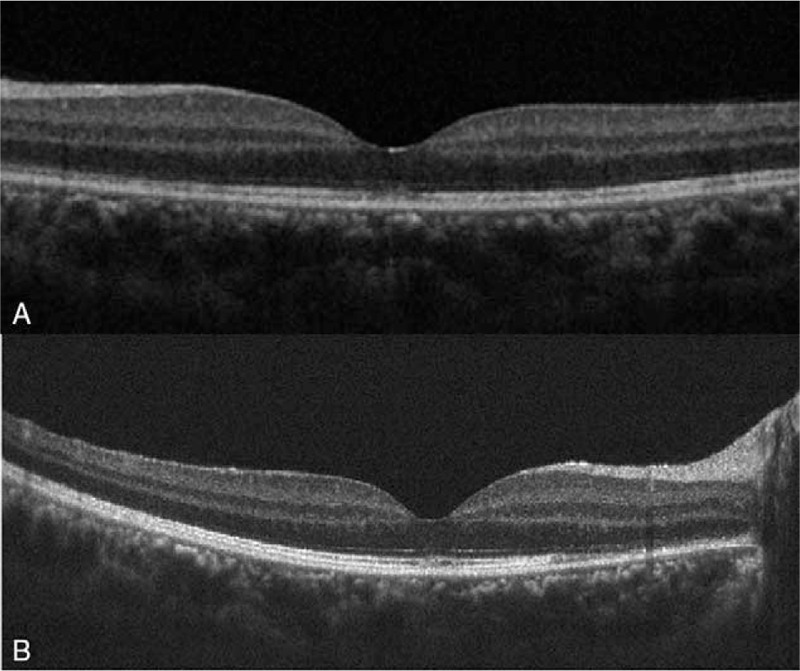
Optical coherence tomography examination (OCT) showed foveal disruption at the ellipsoid layer, also involving interdigitation zone and outer nuclear layer. (A) The first episode occurring in the left eye. (B) The recurrent episode occurring in the right eye.

**Figure 3:**
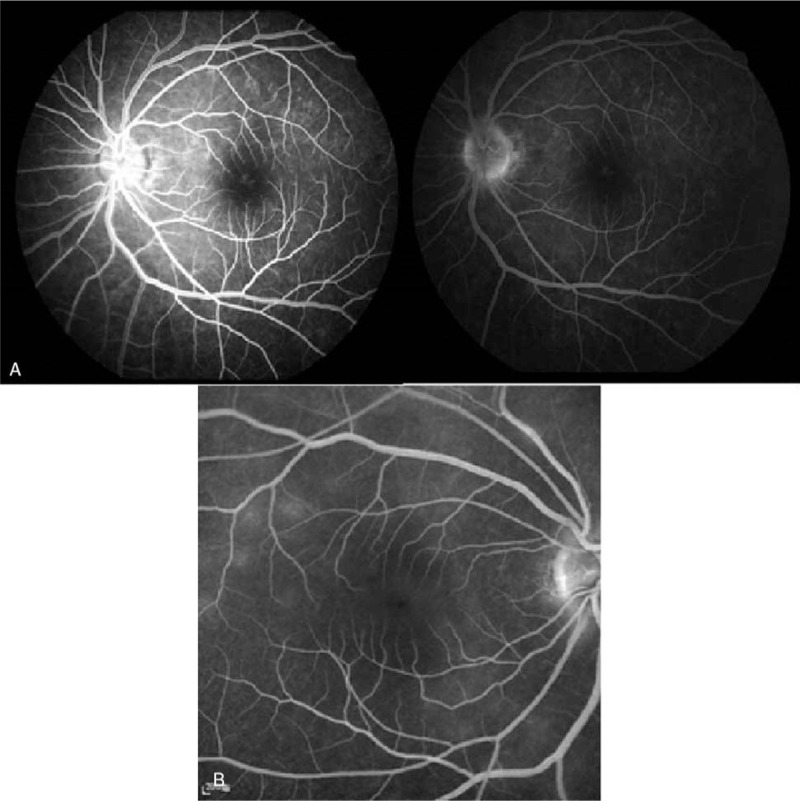
(A) Fluorescein angiography revealed typical “wreath-like” multifocal hyperfluorescent spots of early stage in the left eye during the first episode, (left). Minimally staining lesions in late angiograms were also noted (right). (B) Recurrent episode occurring in the right eye, mild hyperfluorescent spots were shown during late stage.

**Figure 4:**
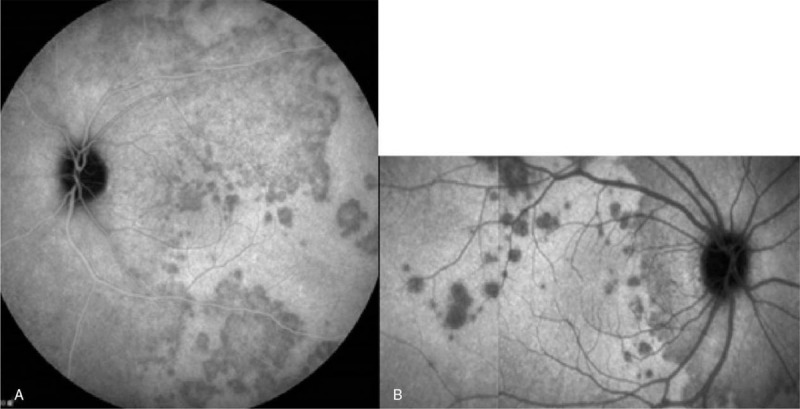
(A) Indocyanine green angiography (ICGA) revealed multiple hypofluorescence dots at the peripapillary area, around fovea and peripheral retina, some were confluent forming larger hypofluorescent zone in the left eye during first episode. (B) Recurrent episode in the right eye, multiple hypofluorescence dots scattered near fovea and were confluent at the peripapillary area.

**Figure 5:**
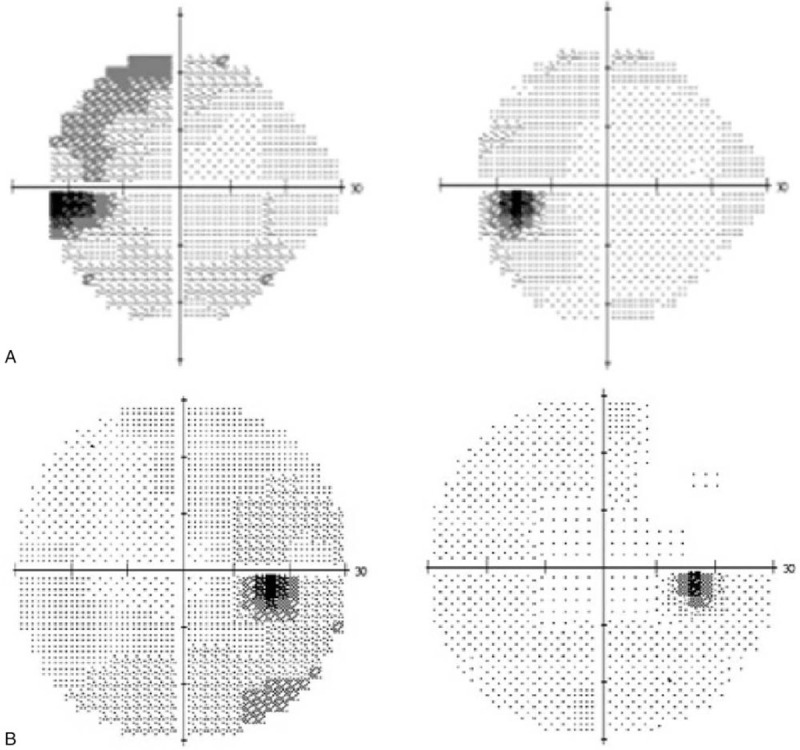
(A) Humphrey visual field testing demonstrated superior and temporal paracentral scotoma with an enlarged blind spot and mean deviation −7.57 decibels in the acute stage of left eye during first episode (left). Follow-up test showed gradually improved with mean deviation −1.94 decibels (right). (B) Recurrent episode in the right eye, visual field testing demonstrated inferior paracentral scotoma with an enlarged blind spot and mean deviation −4.1 decibels (left). Follow-up test showed gradually improved visual field (right).

Fundus photography showed numerous white dots over the macula to the mid-peripheral fundus, with characteristic, foveal, and yellowish granularity (Fig. 1A). OCT examination showed disruption of the EZ and accumulations of hyperreflective material extending through the interdigitation zone, the EZ, and the outer nuclear layer (Fig. 2A). These lesions gradually recovered during the follow-up period. Fluorescein angiography (FAG) revealed typical, “wreathlike,” multifocal, hyperfluorescent, white spots during early stages. Minimally staining lesions in late angiograms were also noted (Fig. 3A). Indocyanine green angiography (ICGA) revealed hypofluorescent dots in both early and mid-phases of the angiogram (Fig. 4A), corresponding with the hyperfluorescent pattern seen in FAG. Late phases of ICGA showed a larger hypofluorescent pattern, and some were confluent, forming a larger hypofluorescence zone. Humphrey 30-2 visual field testing demonstrated a superior and temporal paracentral scotoma with an enlarged blind spot in the acute stage, which gradually improved in the recovery stage (Fig. 5A). Electroretinogram showed markedly decreased scotopic and photopic a-waves and b-waves of the lesion eye. The laboratory data, including blood cell counts, complement C3, C4, erythrocyte sedimentation rates, C-reactive protein, and antinuclear antibodies, were within normal ranges. However, the virus serology immunofluorescent assay test was positive for the VZV immunoglobulin M (IgM) antibody and the herpes simplex virus type 1 (HSV-1) immunoglobulin G (IgG) antibody. Both the VZV IgG antibody and the herpes simplex virus type 2 (HSV-2) IgG antibody were negative. Due to the self-limited nature of MEWDS, we observed the clinical course without intervention. After detailed explanation of the study, the patient had provided informed consent for the publication of the case.

Two years later, the patient experienced persistent flashes of light with acute onset of blurry vision in her right eye following an upper respiratory tract infection. Her previous lesion in the left eye was subjectively fine. The visual acuity of both eyes was 6/5. The IOP was 17 mmHg in the right eye and 18 mmHg in the left eye. Examinations of the anterior chamber and vitreous revealed no cells or flares. As in the initial episode, her fundus photography during recurrence disclosed multiple white dots in the peripheral retina (Fig. 1B). OCT examination showed disruptions of the EZ, with an accumulation of irregularly-shaped, hyperreflective materials at the foveal region (Fig. 2B). FAG revealed mildly hyperfluorescent spots during the late stage, partially corresponding to the white dots seen with fundus photography (Fig. 3B). ICGA showed multiple hypofluorescent dots scattered near the fovea in the late phase (Fig. 4B). Humphrey 30-2 visual field testing demonstrated an inferior and temporal paracentral scotoma with an enlarged blind spot during the initial test (Fig. 5B). Laboratory results disclosed highly elevated titer (1:160) of the Epstein–Barr virus (EBV) capsid antigen (EB-VCA) IgG antibody in the acute phase. The EB-VCA IgM antibody and the VZV IgM antibody were negative in this episode. After 2 months of regular follow-up, the multimodal imaging results and the visual outcome of the patient recovered smoothly, and prior EB-VCA IgG titer decreased fourfold to 1:40 in the recovery stage. Despite the recurrence, this patient still had visual acuity of 6/5 in both eyes.

## 3 Discussion

Recurrent MEWDS may be associated with acute infection of the Herpesviridae family or virus-induced autoimmune inflammatory retinitis. This study reported a chronic, recurrent case of typical MEWDS, with a good visual outcome and complete resolution of retinal white dots and OCT foveal disruption. In this case, during the acute MEWDS phase, concurrent systemic virus infections were confirmed by virus serologic data. These tests were positive for VZV IgM in the first episode and highly elevated for EBV IgG in the recurrent episode.

Current understanding of the pathophysiology of MEWDS is still limited. However, it is widely believed that pathological events in acute MEWDS involve the RPE and the outer segment of photoreceptor cells. Supporting evidence from a recent, multimodal imaging study of early stage MEWDS showed double RPE lines due to fluid leakage and inflammation of the RPE layer.^[7]^ Later, overall disruption of the EZ and the interdigitation layer were observed via OCT. FAG displays classic, wreathlike, hyperfluorescence of retinal lesions, as in this case, coming from dilation of retinal microcirculation due to the inflammation at the middle or deep retinal capillary.^[8]^

The exact mechanism of MEWDS recurrence in this case is still unknown. One possibility of the underlying cause is an infectious etiology.^[9]^ This patient demonstrated a recurrent episode of typical MEWDS presentation in the contralateral eye 2 years after the initial episode. She also suffered from a prodromal flu-like illness before the ocular manifestation of MEWDS in both episodes. During the acute phase of MEWDS, her laboratory serology data was positive for VZV IgM in the first episode and exhibited high EBV IgG titer in the second episode. Both VZV and EBV are members of the Herpesviridae family, sharing a common feature of latency and, later, relapse in the host.^10^,^11^ MEWDS recurrence in this patient over the 2-year period is arguably similar to other cases, in which people experienced relapses of Herpesviridae systemic infections.^[12]^ The latent EBV virus harboring in B cells can be reactivated in vitro by stimulating B-cell receptors. In the present case, this suggests that reactivation in vivo may occur when the infected B-cell responds to unrelated infections.^10^,^11^

Another possible cause of the MEWDS relapse in this patient is a virus-induced, autoimmune reaction. Jampol et al proposed that specific environmental triggers, such as viruses or other pathogens, may interact with susceptive genes, causing predisposed patients to develop specific, autoimmune, inflammatory chorioretinopathy.^[13]^ Based on previous studies of the various pathogens that may be associated with MEWDS,^14^,^15^,^16^,^17^,^18^,^19^ this present research proposes that the pathogens involved play a crucial role in predicting whether MEWDS will relapse. Therefore, the authors hypothesize that, when a patient becomes infected by a member of the Herpesviridae family, the pathogen's ability to be latent and relapse may predispose this patient to suffer from future, recurrent MEWDS. Due to the inability of the host immune system to eliminate this virus completely, viral shedding and EBV-infected cells persist at low levels, approximately 1 in 10,000 to 100,000 memory B cells.^[20]^

The pathogenesis pathway of recurrent MEWDS occurring in the same, or the contralateral, eye is also an issue that warrants further discussion. The authors’ prior study has suggested that, during the MEWDS latent state, RPE may serve as a reservoir for EBV-infected memory B cells.^[18]^ Thus, this work hypothesizes that recurrence in the same eye indicates the reactivation of a latent virus at its original, latent, RPE site. Due to the longevity of memory cells, reactivation of a latent virus may happen a few years after the initial exposure.^[9]^ Conversely, recurrence in the contralateral eye may mean that virus-infected memory B cells hematogenously travel to the retina of the contralateral eye by crossing the blood-retina barrier or by crossing the choriocapillaris and RPE cell barrier.^[18]^ This hypothesis is further supported by the study of Asano et al, who suggested that the pathological, chorioretinal changes in myopic individuals might make their blood-retina barrier more vulnerable to pathogen invasion; hence the higher prevalence of myopia in Japanese patients with MEWDS.^[21]^ In summary, the authors believe that there are different pathogenesis pathways regarding MEWDS relapse in the same eye or the contralateral eye. However, this hypothesis might be challenged by the findings of Li and Kishi,^[22]^ who performed retinal functional tests in MEWDS cases with multifocal and full-field electroretinogram. Their results showed that there was bilateral dysfunction of photoreceptors’ outer segments, despite the unilateral onset of clinical symptoms and the fundus lesions in most patients with MEWDS.^[22]^

The potential limitation of this study lies in the fact that, due to EBV's sensitivity to subtle changes in the immune system, changes in the viral burden or atypical behavior of EBV as detected in serum immunoglobulin levels may be an indirect effect of a compromised immune system, particularly in autoimmune diseases.^[23]^ Anterior chamber paracentesis with polymerase chain reaction of aqueous fluid may be a more specific approach to detect intraocular virus loading.^[24]^ Furthermore, the high percentage of the seropositive population in Taiwan might also create the possibility that the positive serology in MEWDS was found by chance.^[25]^

In conclusion, this research reports that concurrent active virus infections occurred in a case with MEWDS recurrence, and thus, MEWDS recurrence may be associated with acute infection of the Herpesviridae family or virus-induced autoimmune inflammatory retinitis. Future study and comparisons of recurrence patterns for each pathogen may help produce further insights on the pathophysiology of recurrent MEWDS.

## Author contributions

**Investigation:** Yu-Lin Haw, Teng-Chieh Yu.

**Supervision:** Chang-Sue Yang.

**Writing – original draft:** Yu-Lin Haw.

**Writing – review & editing:** Teng-Chieh Yu, Chang-Sue Yang.

## References

[1] Polk TD Goldman EJ White-dot chorioretinal inflammatory syndromes. Int Ophthalmol Clin 1999;39:33–53. 10709581 10.1097/00004397-199903940-00005

[2] Ryan PT Multiple evanescent white dot syndrome: a review and case report. Clin Exp Optom 2010;93:324–9. 20718788 10.1111/j.1444-0938.2010.00507.x

[3] Crawford CM Igboeli O A review of the inflammatory chorioretinopathies: the white dot syndromes. ISRN Inflamm 2013;2013:1–9. 10.1155/2013/783190 PMC3833360 24294536

[4] Jampol LM Sieving PA Pugh D Multiple evanescent white dot syndrome. I. Clinical findings. Arch Ophthalmol 1984;102:671–4. 6721749 10.1001/archopht.1984.01040030527008

[5] Yang CS Wang AG Lin YH Optical coherence tomography in resolution of photoreceptor damage in multiple evanescent white dot syndrome. J Chin Med Assoc 2012;75:663–6. 23245484 10.1016/j.jcma.2012.08.011

[6] Amin HI Optical coherence tomography findings in multiple evanescent white dot syndrome. Retina. 2006; 26:483–4. 16603975 10.1097/01.iae.0000238558.14531.78

[7] Cahuzac A Wolff B Mathis T Multimodal imaging findings in ’hyper-early’ stage MEWDS. Br J Ophthalmol 2017;101:1381–5. 28202480 10.1136/bjophthalmol-2016-309175

[8] Marsiglia M Gallego-Pinazo R Cunha de Souza E Expanded clinical spectrum of multiple evanescent white dot syndrome with multimodal imaging. Retina 2016;36:64–74. 26166804 10.1097/IAE.0000000000000685

[9] Tsai L Jampol LM Pollock SC Chronic recurrent multiple evanescent white dot syndrome. Retina 1994;14:160–3. 8036325 10.1097/00006982-199414020-00009

[10] Odumade OA Hogquist KA Balfour HH Jr Progress and problems in understanding and managing primary Epstein-Barr virus infections. Clin Microbiol Rev 2011;24:193–209. 21233512 10.1128/CMR.00044-10 PMC3021204

[11] Grinde B Herpesviruses: latency and reactivation - viral strategies and host response. J Oral Microbiol 2013;5. 10.3402/jom.v5i0.22766 PMC3809354 24167660

[12] Benedetti J Corey L Ashley R Recurrence rates in genital herpes after symptomatic first-episode infection. Ann Intern Med 1994;121:847–54. 7978697 10.7326/0003-4819-121-11-199412010-00004

[13] Jampol LM Becker KG White spot syndromes of the retina: a hypothesis based on the common genetic hypothesis of autoimmune/inflammatory disease. Am J Ophthalmol 2003;135:376–9. 12614757 10.1016/s0002-9394(02)02088-3

[14] Fine L Fine A Cunningham ET Jr Multiple evanescent white dot syndrome following hepatitis a vaccination. Arch Ophthalmol 2001;119:1856–8. 11735803 10.1001/archopht.119.12.1870

[15] Baglivo E Safran AB Borruat FX Multiple evanescent white dot syndrome after hepatitis B vaccine. Am J Ophthalmol 1996;122:431–2. 8794720 10.1016/s0002-9394(14)72074-4

[16] Goyal S Nazarian SM Thayi DR Multiple evanescent white dot syndrome following recent influenza vaccination. Can J Ophthalmol 2013;48:e115–116. 24093200 10.1016/j.jcjo.2013.03.002

[17] Cohen SM Multiple evanescent white dot syndrome after vaccination for human papilloma virus and meningococcus. J Pediatr Ophthalmol Strabismus 2009;25:1–3. 10.3928/01913913-20090616-01 19645392

[18] Yang CS Hsieh MH Su HI Multiple evanescent white dot syndrome following acute epstein-barr virus infection. Ocul Immunol Inflamm 2017;27:1–7. 10.1080/09273948.2017.1371763 29020489

[19] Yang JS Chen CL Hu YZ Multiple evanescent white dot syndrome following rabies vaccination: a case report. BMC Ophthalmol 2018;18:312. 30526550 10.1186/s12886-018-0968-y PMC6286608

[20] Laichalk LL Hochberg D Babcock GJ The dispersal of mucosal memory B cells: evidence from persistent EBV infection. Immunity 2002;16:745–54. 12049725 10.1016/s1074-7613(02)00318-7

[21] Asano T Kondo M Kondo N High prevalence of myopia in Japanese patients with multiple evanescent white dot syndrome. Jpn J Ophthalmol 2004;48:486–9. 15486773 10.1007/s10384-004-0107-6

[22] Li D Kishi S Restored photoreceptor outer segment damage in multiple evanescent white dot syndrome. Ophthalmology 2009;116:762–70. 19344825 10.1016/j.ophtha.2008.12.060

[23] Thorley-Lawson DA Gross A Persistence of the Epstein-Barr virus and the origins of associated lymphomas. N Engl J Med 2004;350:1328–37. 15044644 10.1056/NEJMra032015

[24] Rath S Mohan N Basu S The diagnostic utility of anterior chamber paracentesis for polymerase chain reaction in anterior uveitis. Am J Ophthalmol 2013;156:847. 10.1016/j.ajo.2013.07.003 24053895

[25] Chen CY Huang KY Shen JH A large-scale seroprevalence of Epstein-Barr virus in Taiwan. PLoS One 2015;10:e0115836. 25615611 10.1371/journal.pone.0115836 PMC4304788

